# Delayed right chylothorax after left blunt chest trauma: a case report

**DOI:** 10.1186/s13256-017-1250-2

**Published:** 2017-04-10

**Authors:** Jonggeun Lee, Jeong Su Cho, Hoseok I, Yeong Dae Kim

**Affiliations:** 1 Department of Thoracic and Cardiovascular Surgery, Jeju National University Hospital, Jeju National University School of Medicine, Jeju, South Korea; 2 Department of Thoracic and Cardiovascular Surgery, Pusan National University School of Medicine, Medical Research Institute, Pusan National University Hospital, 179 Gudeok-Ro, Seo-Gu, Busan, 602-739 Republic of Korea

**Keywords:** Delayed chylothorax, Chylothorax, Thoracic duct, Chest trauma

## Abstract

**Background:**

Chylothorax is a disease that has various causes such as neoplasm, infection, post-surgery trauma, congenital, and venous thrombosis. In approximately 15% of cases of chylothorax, the exact cause is unknown. We report a case of delayed occurrence of right chylothorax in a patient who had multiple segmental ribs fracture on his left side.

**Case presentation:**

A 70-year-old Asian man had a “rollover” accident in which the cultivator he was driving overturned. He presented to our hospital with the main complaint of severe dyspnea. On chest computed tomography, multiple ribs fracture from the first to the eighth rib of the left side of his chest and left-sided hemopneumothorax were presented, but there was no evidence of fracture in the right side of his chest.

After closed thoracostomy, an emergency operation for open reduction of fractured ribs was performed. On the fifth postoperative day, tubal feeding was performed. On the next day, a plain chest X-ray image showed pleural effusion of the right side of his chest. After insertion of a small-bore chest tube, 3390 ml of fluid for 24 hours was drained. The body fluid analysis revealed triglycerides levels of 1000 mg/dL, which led to a diagnosis of chylothorax. Although non-oral feeding and total parenteral nutrition were sustained, drain amount was increased on the fifth day. Surgical treatment (thoracoscopic thoracic duct ligation and pleurectomy) was performed in the early phase. The right chest tube was removed on the 14th postoperative day after the effusion completely resolved and he was uneventfully discharged.

**Conclusions:**

In this case, as our patient was in old age and had multiple traumas, surgical treatment for chylothorax needed to be performed in the early phase.

## Background

Chylothorax occurs as the result of laceration, rupture, or obstruction in the thoracic duct or its branches. Common causes of chylothorax include neoplasm, trauma, infection, and venous thrombosis [1–3] and it has been known that nearly 30% of cases are accompanied by fatal complications such as heart failure, malnutrition, and immunodepression [2]. The exact cause of chylothorax is unknown in approximately 15% of cases. We intend to introduce the progress and treatment of delayed chylothorax that occurred contralaterally after trauma.

## Case presentation

**Fig. 1:**
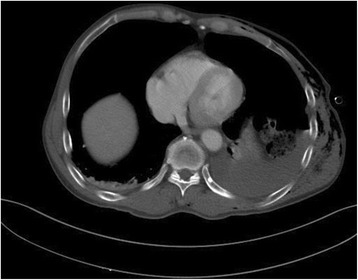
Chest computed tomography scan taken at admission revealed multiple ribs fracture and hemopneumothorax, left

A 70-year-old Asian man had a “rollover” accident in which the cultivator he was driving overturned. Due to the accident, he presented with hemothorax and flail chest on the first to eighth ribs on the left side of his chest. A chest computed tomography (CT) scan taken at an emergency room (ER) revealed multiple ribs fracture and hemopneumothorax with subcutaneous emphysema of the left side of his chest and atelectasis of the right side of his chest (Fig. 1).

**Fig. 2:**
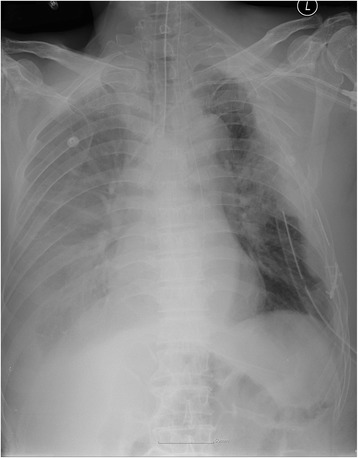
Chest X-ray on fifth postoperative day after open reduction of multiple ribs fracture on left side

**Fig. 3:**
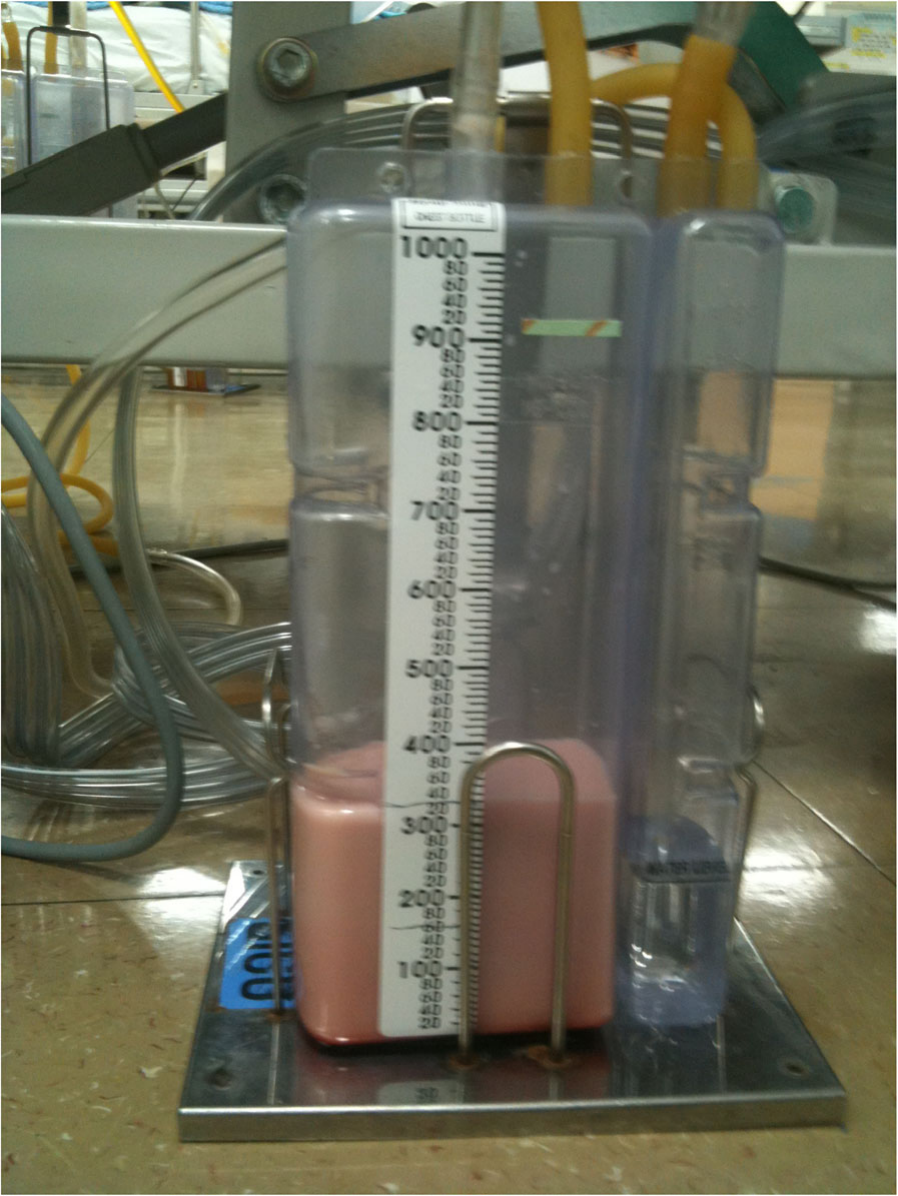
The color of drained fluid

**Fig. 4:**
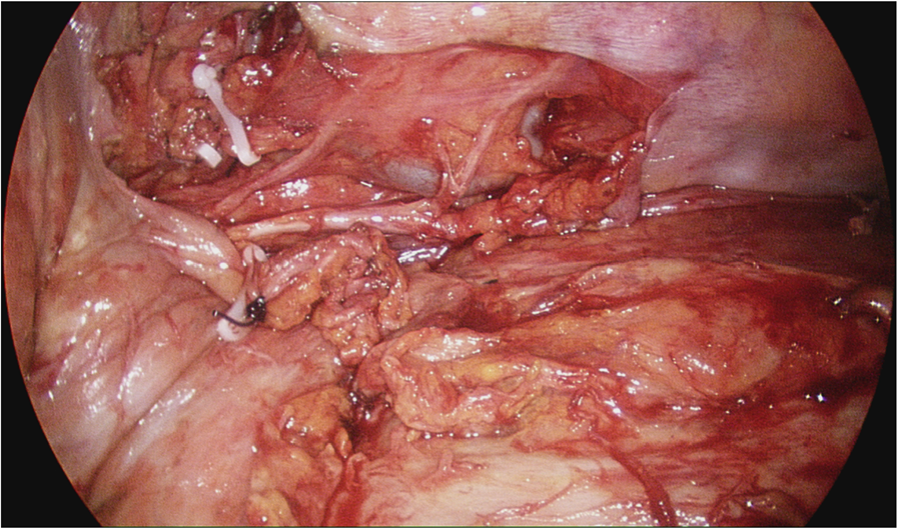
Thoracoscopic intraoperative view

**Fig. 5:**
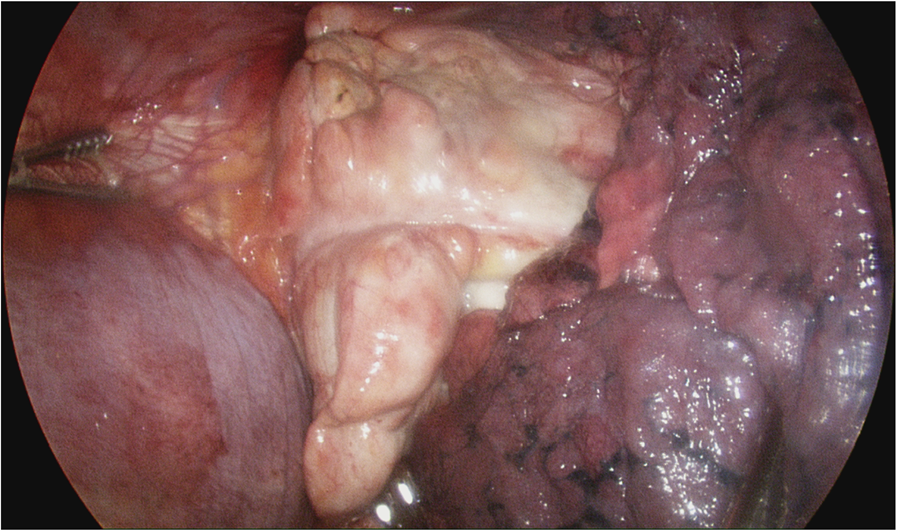
Thoracoscopic mass ligation of thoracic duct, right

He was transferred to the ER of our hospital. The hemothorax and the condition of his chest wall indicated surgery and we performed emergency surgery of open reduction for fractured ribs and primary repair for lacerated lung. Due to the flail chest, he needed the support of a mechanical ventilator for stable respiration. As part of postoperative ventilator care, tubal feeding was started on the fourth postoperative day (POD). On the next day (fifth POD), a chest X-ray showed a large amount of right pleural effusion (Fig. 2). A small-bore chest catheter (16 gauge) was placed in right pleura. Approximately 880 ml of pleural fluid was initially drained and in total 3390 ml was drained for 24 hours. The color of the fluid changed from pinky to creamy (Fig. 3). Laboratory analysis of the fluid showed high triglycerides (>1000 mg/dl) and low total cholesterol levels (6 mg/dl). The amount of drainage increased to 3000 to 4000 ml/day on the fifth day after the initiation of non-oral feeding (NPO) and total parenteral nutrition (TPN). Because of the extremely large amount of drained fluid, thoracoscopic thoracic duct ligation and pleurectomy on the right was performed the next day (sixth POD). We decided to do early-phase surgery because our patient was an elderly patient with trauma and a long period of fasting would be likely to cause problems with nutrition. His chest was explored, but no obvious leak was identified. The pleura between his aorta and esophagus was dissected at the base of his diaphragm and the dissection continued toward his right-sided thorax between his posterior aorta and the vertebral bodies. There was a mass-like lesion combined with pericardial fat and chyle (Figs. 4 and 5). After this finding, surgical ligation of his thoracic duct was decided and a pleurectomy was performed to reduce the risk of recurrent malignant effusion. Immediately postoperatively, the drainage changed to serosanguineous fluid, without any evidence of chyle. Two days after the operation, the drains were serous and the amount of drainage was reduced to less than 100 ml/day. He started a fat-free diet after surgery and then his diet was changed to fat content and medium-chain triglycerides diet.

**Fig. 6:**
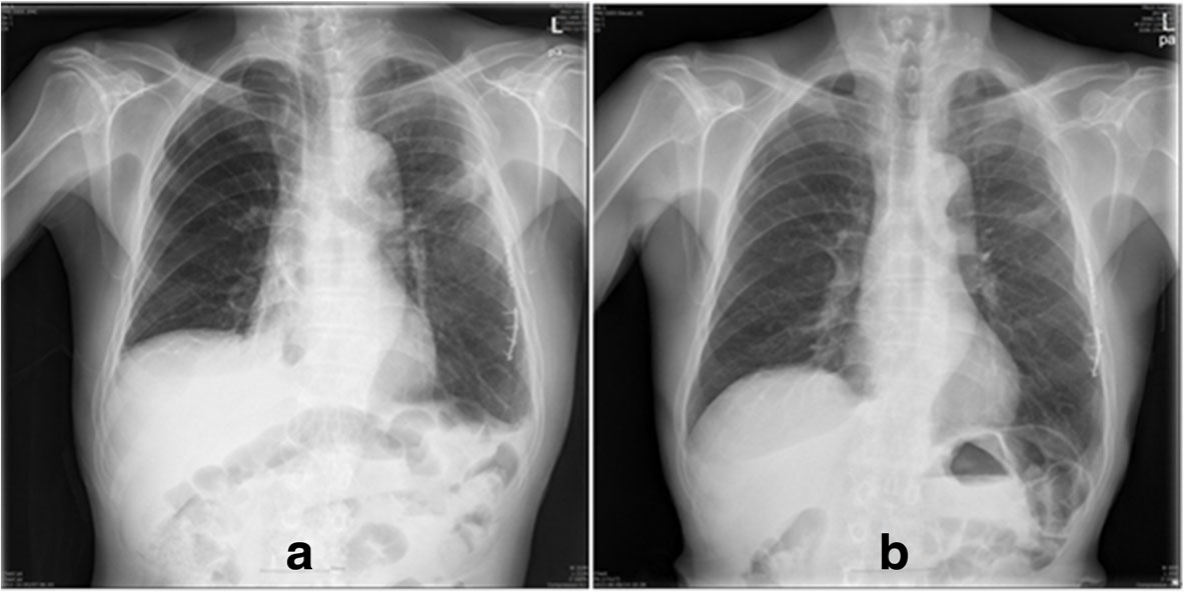
**a** Chest X-ray at discharge day. **b** Chest X-ray at 1 year after treatment

Although the removal of the chest tube was indicated after surgery, the removal of the chest tube was delayed because of the risk of malnutrition. The chest tube was removed on the 14th POD after amount and characteristics of drainage were examined and the effusion had completely resolved; he was then uneventfully discharged. He is on follow-up without any sign of recurrence a year after surgery (Fig. 6).

## Discussion

Traumatic injury to the thoracic duct may occur after cervical, thoracic, or abdominal surgical procedures or as a result of penetrating or blunt trauma. Chylothorax is a rare complication of blunt chest trauma, as the thoracic duct is generally well protected by the spine posteriorly and mediastinal contents anteriorly. Although thoracic or lumbar spinal injury is a common occurrence after blunt chest trauma, very few patients will have an associated chylothorax. The most common mechanism of injury to the thoracic duct after blunt trauma appears to be sudden hyperextension of the spine [4, 5]. This results in rupture of the duct due to stretching over the vertebral bodies or a shearing of the duct by the right crus of the diaphragm, even without evidence of vertebra fracture or dislocation [6]. In this case, our patient had no evidence of vertebra injury, but delayed right chylothorax may have occurred after spine hyperextension not accompanied by vertebra fracture.

The loss of chyle and lymph into the pleural space can lead to loss of water, electrolytes, proteins, immunoglobulins, fat, and essential vitamins. Patients are usually able to compensate in the early stages but in advanced cases there may be signs and symptoms of malnutrition and hypovolemia. Acidosis, hyponatremia, and hypocalcemia are the most common abnormalities [7]. Continued loss of proteins, immunoglobulins, B lymphocytes, and T lymphocytes into the pleural space can lead to immunosuppression [8].

**Fig. 7:**
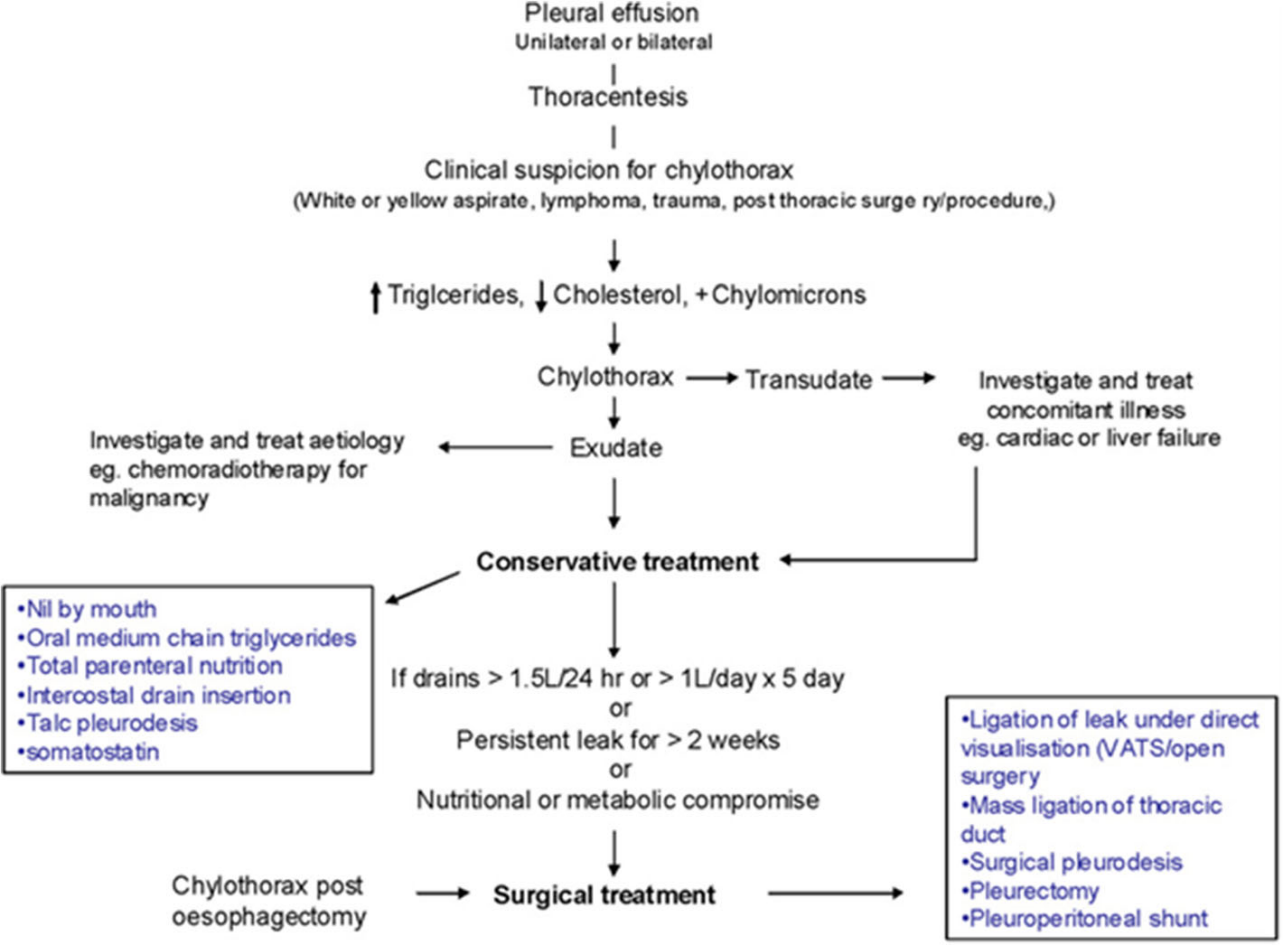
Management pathway of chylothorax. *VATS* video-assisted thoracoscopic surgery

In general, the goal of initial treatment of chylothorax is to facilitate re-expansion of lung by performing drainage as conservative management, to prevent dehydration, and to reduce chyle formation by supplying proper nutrition. Treatment can be divided to conservative management [9] and surgical management [10] according to the cause of chylothorax and amount of drainage (Fig. 7). Even though the algorithm of chylothorax treatment is controversial, the treatment of chylothorax includes conservative medication such as diet, radiotherapy, chest tube drainage, and chemical pleurodesis and surgical methods such as ductal ligation, pleura-peritoneal shunt, and pleura-venous shunt [10–13]. In addition, there are some institutions that use octreotide infusions as a way to decrease the output of chylous effusions [14, 15]. Conservative measures have achieved as high as an 88% success rate [16]. Chyle has an irritating nature which actually promotes pleurodesis and in half of patients the leak will stop spontaneously.

In 50% of the cases, non-surgical treatment for 10 to 14 days cured the disease [17]. If the conservative treatment fails so much that the drainage has accelerated in daily output to 200 to 500 mL per day for 1 to 3 weeks, it may be possible to consider an operative technique such as thoracic duct ligation or thoracic duct embolization. Surgery enables a reduction in the period of being hospitalized and prevents complications that may have occurred by chylothorax [10]. However, there is no consensus on the length of time before surgical therapy should be considered in a patient whose drainage has significantly decreased. Although 4 further weeks of chest drainage has been suggested empirically [18], some have favored a more aggressive approach, with immediate thoracotomy and thoracic duct ligation if the leak has not resolved after 2 weeks of observation [5].

But, chylothorax can have an impact on respiratory distress and chronic depletion of chyle [19], and particularly old patients could become vulnerable to infections and malnutrition, particularly in the postoperative period [20]. In our case, the patient recovered without any clinical symptoms of chyle loss due to early operation.

## Conclusions

In this case, as our patient was in old age and had multiple traumas, we considered that fasting for a long time would have a bad impact on his recovery. For this reason, surgical treatment was performed in the early phase and he was discharged from our hospital without problem.
